# Combined creatine and β-hydroxy-β-methylbutyrate supplementation with integral conditioning exercise enhances functional performance and metabolic health in physically active older adults: A randomized controlled crossover trial

**DOI:** 10.1007/s40520-025-03312-0

**Published:** 2026-01-09

**Authors:** Rafael Ramos-Hernández, Natalia Busto, Álvaro Miguel-Ortega, María Martínez-Ferrán, Mirian Santamaría-Peláez, Miriam Saiz-Rodríguez, Juan Mielgo-Ayuso

**Affiliations:** 1 Faculty of Health Sciences, University of Burgos (UBU), Burgos, 09001 Spain; 2 IAFIV Research Group, University of Burgos (UBU), Burgos, 09001 Spain; 3 Faculty of Education, Alfonso X ‘El Sabio’ University (UAX), Madrid, 28691 Spain; 4 Working Group on Nutrition for Exercise and Sport, Spanish Nutrition Society “SEÑ”, Madrid, 28010 Spain

**Keywords:** Creatine monohydrate, Β-hydroxy-β-methylbutyrate, Older adults, Multicomponent training, Functional mobility, Metabolism, Endothelial function

## Abstract

**Background:**

Combined creatine monohydrate (CRE) and β-hydroxy-β-methylbutyrate (HMB) supplementation may counteract age-related declines in functional capacity, yet evidence in physically active older adults is scarce.

**Objective:**

To investigate the effects of six weeks of CRE + HMB supplementation integrated with a supervised multicomponent exercise program on functional performance, metabolic efficiency, and physiological health in older adults.

**Methods:**

Thirty physically active adults aged ≥ 60 years (20 men, 10 women) completed a randomized, double-blind, placebo-controlled crossover trial involving two 6-week intervention phases (CRE + HMB or placebo), separated by a 3-week washout. The exercise program (4 sessions/week) combined strength, endurance, and coordination training. Functional tests (4-m gait speed, 5-repetition sit-to-stand, Timed Up and Go, 400-m walk), metabolic indices, and cardiopulmonary and inflammatory markers were assessed pre- and post-intervention.

**Results:**

CRE + HMB significantly improved gait speed, sit-to-stand, TUG, and 400-m walk (*p* < 0.05), with large effect sizes (η²*p* = 0.15–0.29). Basal metabolic rate and metabolic rate index increased, while visceral adiposity showed favorable trends. Women exhibited reductions in diastolic blood pressure and higher expiratory strength; men showed a transient rise in endothelial protein C receptor (EPCR). No period, sequence, or carryover effects were detected.

**Conclusions:**

Six weeks of CRE + HMB supplementation integrated with supervised multicomponent training enhanced mobility, metabolic efficiency, and selected physiological outcomes in physically active older adults. This strategy represents a safe, feasible, and practical approach to sustain functional independence and metabolic health with aging.

**Supplementary Information:**

The online version contains supplementary material available at 10.1007/s40520-025-03312-0.

## Introduction

Aging is accompanied by a progressive decline in physical function—reductions in muscle strength, balance, mobility, and endurance—that increases the risk of falls, frailty, hospitalization, and loss of independence [1–3]. Preserving functional capacity is therefore a public health priority in older adults [4]. In parallel, aging entails marked metabolic changes, including lower basal metabolic rate (BMR), which are linked to insulin resistance, chronic low-grade inflammation, and higher cardiometabolic risk [5–7]. Physical and metabolic impairments are tightly interrelated [8, 9], creating a cycle of decline that accelerates the aging process. Consensus statements such as EWGSOP2 highlight gait speed and muscle strength as primary indicators of sarcopenia, reinforcing the importance of targeting these domains in intervention studies [1].

Exercise is the cornerstone intervention to preserve muscle and function in aging, via anabolic signaling, neuromuscular adaptations, and broad performance gains [4, 10]. However, anabolic resistance blunts the full adaptive response in many older adults [11]. Multicomponent Integral Physical Conditioning (IPC) programs—integrating resistance, endurance, balance, mobility/flexibility, and coordination—consistently improve functional fitness, fall-related outcomes, and mobility in older populations [12–15], but may not fully prevent declines in metabolic efficiency or adequately modulate low-grade inflammation on their own [16, 17].

Targeted nutrition can augment training adaptations. Creatine monohydrate (CRE) supports high-energy phosphate availability during intermittent, high-intensity efforts and may enhance anabolic signaling [18–21], whereas β-hydroxy-β-methylbutyrate (HMB) attenuates proteolysis and supports membrane integrity, potentially reducing exercise-induced muscle damage and favoring net protein balance [22–26]. Their complementary mechanisms—CRE enhancing the capacity for powerful movements and HMB supporting recovery and preservation—provide a biologically plausible synergy for improving functional mobility, metabolic efficiency, and overall training adaptation in aging [27]. Synergistic improvements in strength and lean mass have been reported in young and endurance athletes with CRE + HMB [22, 27, 28]. Recently, our group demonstrated that this combined supplementation, when integrated with multicomponent exercise, significantly improved functional strength (handgrip, back dynamometry, and muscular endurance tests) in physically active older adults [29].

However, evidence in this population remains scarce, particularly regarding mobility, metabolic efficiency, and vascular health. To date, no randomized crossover trials have simultaneously examined functional performance, metabolic parameters, and endothelial biomarkers in physically active older adults, a demographic increasingly interested in maintaining autonomy and delaying age-related decline [30–32]. Accordingly, we prioritized sensitive functional outcomes—4-m gait speed, 5-repetition sit-to-stand (5R-STS), Timed Up and Go (TUG), and the 400-m walk—alongside the Short Physical Performance Battery (SPPB) to ensure comparability with international standards.

To comprehensively characterize the intervention’s effects, we also included metabolic and inflammatory biomarkers. Endothelial Protein C Receptor (EPCR) was selected as a novel vascular–inflammatory marker due to its dual role in endothelial protection and coagulation balance [33, 34]. Metabolic parameters including BMR and visceral adiposity were assessed to evaluate potential shifts in energy metabolism and body composition.

The 6-week supplementation period was chosen based on evidence that moderate daily doses of CRE and HMB (3 g/day each, without loading) elicit measurable neuromuscular and metabolic adaptations within this timeframe [28, 29, 35]. A 3-week washout was implemented, justified by the pharmacokinetic profile of CRE—characterized by gradual saturation and faster clearance under low-dose regimens [ati^36^, ^37^]—and supported by previous findings showing no carryover effects in comparable protocols [29]. The randomized crossover design was selected for its superior efficiency and within-subject control, minimizing interindividual variability in this homogeneous, active population [38].

Therefore, the primary objective of this double-blind, placebo-controlled crossover trial was to determine whether six weeks of combined CRE + HMB supplementation integrated with a standardized multicomponent IPC program improves functional performance (4-m gait speed, 5R-STS, TUG , and 400-m walk) in physically active older adults.

Secondary objectives were to assess changes in metabolic/physiological indicators (BMR, visceral fat index, blood pressure, heart rate, Peripheral Oxygen Saturation (SpO₂), and maximal expiratory pressure (MEP)) and inflammatory status (EPCR). Exploratory analyses were pre-specified to examine sex-specific responses, given known differences in muscle metabolism and adaptation to combined exercise–nutrition interventions in older adults [39, 40]. Based on prior mechanistic and experimental evidence [18–28, 41], we hypothesized that CRE + HMB supplementation combined with IPC would lead to significantly greater improvements in functional, metabolic, and physiological outcomes than placebo.

## Materials and methods

### Study design and participants

This randomized, double-blind, placebo-controlled crossover trial was conducted with 30 physically active older adults (62.7 ± 5.3 years; range: 60–82; 20 men, 10 women) recruited from senior centers and community sports programs in Tenerife, Spain. The study protocol was approved by the University of Burgos Ethics Committee (IR 24/2023) and prospectively registered at ClinicalTrials.gov (NCT05951439). All participants provided written informed consent.

#### Participants

Forty volunteers were initially screened. Seven were excluded for not meeting inclusion criteria or declining to discontinue other supplements. Three participants withdrew for personal reasons unrelated to the intervention, resulting in a final sample of 30 and a low attrition rate of 10%. No withdrawals were due to adverse effects from supplementation or exercise.

**Inclusion criteria** were: age ≥ 60 years, physical independence, and engagement in ≥ 150 min/week of moderate-intensity physical activity. **Exclusion criteria** included uncontrolled chronic disease or advanced stages of cardiovascular, metabolic, renal, hepatic, or musculoskeletal disorders (e.g., NYHA Class III–IV heart failure, HbA1c > 8.5%, CKD stage ≥ 4, severe osteoarthritis). Individuals with stable, mild-to-moderate conditions were included with medical clearance.

#### Sample size justification and outcomes

An a priori power analysis was conducted using G*Power 3.1.9.7 (F tests, repeated measures, within factors) to estimate the minimum sample size required for detecting a medium within-subject effect size (f = 0.25, equivalent to η²*p* ≈ 0.06) with α = 0.05, power (1 − β) = 0.80, two measurements, *r* = 0.70, and nonsphericity ε = 1.0. This yielded a required total sample size of *N* = 21.

For comparison, a parallel-group design (repeated-measures ANOVA, within–between interaction; same parameters) would require approximately *N* = 54 participants to achieve equivalent power. The 2 × 2 randomized crossover structure used here is known to increase statistical efficiency by exploiting within-subject variance and balancing period and sequence effects, thereby reducing the required sample size without compromising power [38]. Recruitment of 40 participants accounted for potential attrition, and the 30 completers provided adequate statistical power, as the observed partial η² values (0.17–0.42) for the primary functional outcomes consistently exceeded the medium effect assumed in the a priori analysis. This efficiency gain, typically associated with a 30–50% reduction in intra-subject error variance compared with parallel designs, further supports the adequacy of the achieved sample size.

Primary outcomes, aligned with EWGSOP2 recommendations [1], were a family of functional performance measures: 4-m gait speed, 5R-STS, TUG, and the 400-m walk test. **Secondary outcomes** included metabolic parameters (BMR, visceral fat index), physiological measures (blood pressure, SpO₂, MEP), and inflammatory status (EPCR). Exploratory analyses examined sex-specific responses.

**Randomization**,** Blinding**,** and Washout**: Participants were stratified by sex and randomly allocated (1:1) to one of two treatment sequences (CRE + HMB→Placebo or Placebo→CRE + HMB) using a computer-generated sequence by an independent researcher. All participants, researchers, and outcome assessors were blinded to group allocation. Supplements and placebos were identical in appearance and packaging.

The 3-week washout period was rigorously justified: (I) The use of a maintenance-dose CRE regimen (3 g/day) without a loading phase leads to gradual saturation and more rapid clearance compared to high-dose protocols [42, 43]; (II) Our previous crossover trial in an identical population using the same supplementation and washout found no statistical carryover effects [44]; and (III) Formal testing in the present study (T1 vs. T3 comparisons and inclusion of sequence/period terms in statistical models) confirmed the absence of residual effects (Table 4).

#### Study timeline and assessments

The study comprised three stages over ~ 21 weeks.

- **Stage 1**: 6-week intervention (Baseline T1; Post-intervention 1, T2).
- **Stage 2**: 3-week washout (Pre-intervention 2, T3).
- **Stage 3**: 6-week intervention (Post-intervention 2, T4).

For the primary crossover analysis, T1 and T3 were pooled as the pre-intervention (PRE) value, and T2 and T4 were pooled as the post-intervention (POST) value. The initial baseline (T1) was used exclusively for verifying group equivalence prior to any intervention. All assessments were conducted by trained evaluators, blinded to treatment allocation, under standardized conditions.

**Fig. 1:**
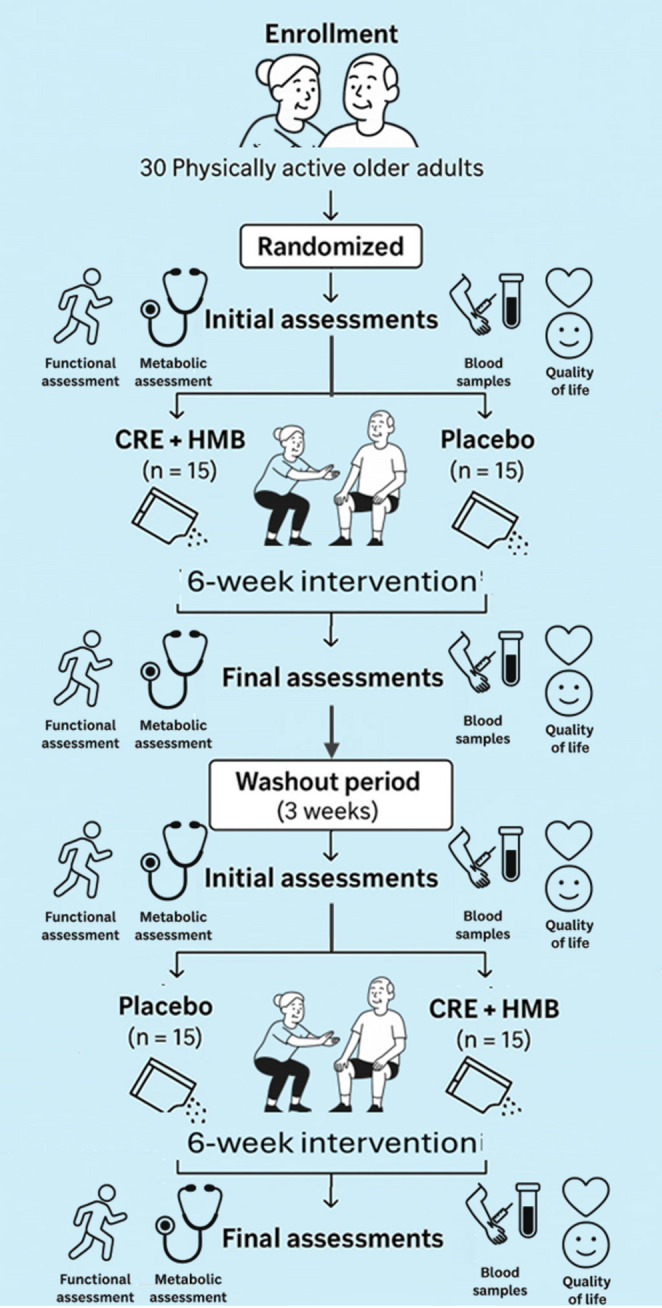
Schematic overview of the crossover study design, detailing baseline metabolic, functional, physiological, and quality of life assessments, followed by two 6-week intervention phases with either CRE plus HMB supplementation or placebo and an integral physical conditioning exercise program separated by a 3-week washout period. After the washout, participants switched to alternate interventions to complete the crossover sequence

The overall study design is summarised in Fig. 1.

### Supplementation protocol

During each 6-week intervention phase, participants received either 3 g/day of CRE combined with 3 g/day of HMB (provided as calcium HMB; HMB-Ca) or an isocaloric placebo consisting of 6 g/day of inulin. All supplements were provided in identical, opaque sachets containing 6 g of powder per day to ensure complete blinding [18, 35–37, 45–47].

Participants were instructed to dissolve one sachet daily in yogurt or fruit juice and to consume it approximately 30 min before bedtime, a schedule previously shown to optimize gastrointestinal tolerance and adherence in older adults [29]. All nutritional guidance and supplementation instructions were delivered individually by a registered dietitian-nutritionist. Supplement packages containing 42 sachets (covering each 6-week phase) were distributed at the T1 and T3 assessments.

Compliance was monitored weekly through participant logs, direct supervision during exercise sessions, and sachet counts at each assessment. Reported adherence exceeded 95%, and no supplementation-related adverse events were observed.

The selected dosage and duration were based on previous studies demonstrating the safety and efficacy of 3 g/day of CRE and 3 g/day of HMB in both older and athletic populations [28, 35, 45]. This moderate, loading-free dosing strategy has been shown to produce measurable improvements in functional strength, metabolic efficiency, and recovery capacity within 6–8 weeks, while maintaining excellent tolerability and compliance [44].

#### Placebo selection and rationale

Inulin was selected as the placebo primarily due to its isocaloric value and its near-identical visual and textural properties to the active supplement powder, which was critical for maintaining blinding.

Crucially, the dose of 6 g/day was strategically chosen to be below the threshold for significant systemic effects. The review by Slavin (2013) [49] indicates that doses of inulin-type fructans typically need to exceed 8–10 g/day to consistently demonstrate prebiotic efficacy and associated systemic changes. Furthermore, a recent RCT in a clinical population by Vaghef-Mehrabani et al. (2023) [48] administered 10 g/day of inulin for 8 weeks and found no significant effects on key inflammatory biomarkers (including CRP, TNF-α, and IL-6) compared to a maltodextrin placebo. Our lower dose (6 g/day) and shorter duration (6 weeks) therefore fall within a range demonstrated in the literature to be unlikely to produce confounding anti-inflammatory or ergogenic effects.

This, combined with the absence of any supplement-related withdrawals or reported gastrointestinal disturbances in our study, supports its suitability as a physiologically inert comparator in this specific context [48, 49].

While inulin exhibits prebiotic activity at higher doses or longer durations, the 6-week intervention period and moderate dose used in this study were insufficient to produce systemic effects that could confound the outcomes.

#### Washout period considerations

The 3-week washout period was justified by the pharmacokinetic profiles of both compounds. CRE and HMB have short plasma half-lives (approximately 3 h and 2.5 h, respectively) [36, 37]. More importantly, under moderate daily dosing (3 g/day) without a loading phase, intramuscular creatine stores decline by approximately 1.6–1.7% per day, returning to near-baseline levels within 3–4 weeks of cessation [42, 43, 50]. This evidence, combined with formal statistical testing confirming no carryover effects (see Sect. 2.3), supports the adequacy of the 3-week washout for this supplementation protocol.

### Nutritional monitoring

Participants were instructed to maintain their habitual dietary patterns throughout the study. In line with current nutritional guidelines for healthy ageing and exercise performance in older adults [40], they were specifically encouraged to achieve a daily protein intake of more than 1.2 g/kg/day and an energy intake of approximately 35–40 kcal/kg/day. These recommendations were reinforced during familiarisation sessions and periodically reviewed by a registered dietitian–nutritionist.

Dietary intake was monitored using a validated, semi-quantitative Food Frequency Questionnaire (FFQ) administered at the end of each 6-week intervention phase [51]. The FFQ assessed 24 grouped food categories (dairy, meat, fish, cereals, fruits, vegetables, healthy fats, beverages, and processed foods), providing a comprehensive overview of habitual energy and macronutrient intake. This instrument has demonstrated good test–retest reliability in Spanish adults (*r* ≈ 0.69–0.81 for energy and macronutrients) and sensitivity to dietary variation while minimizing participant burden [51].

#### Rationale for dietary assessment method

The FFQ was chosen to confirm dietary stability between phases—primary for a crossover design—rather than to capture short-term absolute intake with high resolution. Although 24 h recalls or 3-day records can provide more detail acutely, they impose higher burden and are more susceptible to day-to-day variability and recall bias in longer interventions with older adults [52, 53]. In contrast, the FFQ provides a valid and feasible estimate of habitual patterns, supporting compliance and internal validity when each participant serves as their own control.

Analysis of FFQ data showed no significant differences in total energy or macronutrient intake (including protein, carbohydrate, and fat) between the CRE + HMB and placebo phases (all *p* > 0.05), supporting dietary stability across the trial.

### Integral physical conditioning program (IPC)

All participants followed the same individualized IPC programme during both intervention phases. The IPC was designed to enhance overall physical fitness and counteract age-related declines in muscle strength, power, endurance, balance, and mobility, in accordance with the American College of Sports Medicine (ACSM) guidelines for exercise prescription in older adults [54]. The programme consisted of four supervised 60-minute sessions per week, with overall adherence exceeding 90%. The training model integrated multiple components within each session to reflect the multidimensional nature of daily physical tasks, a strategy demonstrated to produce superior functional transfer compared to single-modality protocols [55, 56].

#### Periodization and structure

The IPC was divided into two 6-week training blocks, separated by the 3-week washout. Each block comprised two mesocycles of three microcycles (weeks). The initial microcycle served as a familiarization phase with higher volume and lower intensity to ensure safe neuromuscular adaptation. Subsequent microcycles incorporated progressive increases in load, technical demand, and exercise complexity. This periodized approach, prioritizing motor learning and gradual overload, is recognized for maximizing neuromuscular and functional gains while maintaining safety in older populations [55, 56].

#### Session structure

Each session followed a standardized three-part structure.

**Warm-up (5–12 min)**: Progressive mobility, balance, and coordination drills (e.g., joint mobilisation, dynamic stretching, gait patterns).
**Main Part (20–50 min)**: Integration of several training modalities:
(a) **Strength Training**: Multi- and single-joint exercises for all major muscle groups. Loads progressed from 50 to 60% of the estimated one-repetition maximum (1RM) in introductory weeks to 60–90% 1RM in advanced phases, for 2–4 sets of 6–12 repetitions.
(b) **Power Training**: Explosive, high-velocity movements (e.g., medicine ball throws, plyometric jumps), progressing from 20 to 60% to ≥ 60–80% 1RM.
(c) **Multicomponent Circuits (MCC)**: 6–12 station circuits combining strength, cardiovascular endurance, balance, coordination, and agility tasks with minimal rest.
(d) **High-Intensity Interval Training (HIIT)**: Alternating bouts of 20–60 s at 80–100% of training heart rate (THR) or ≥ 80% 1RM with 20–90 s active recovery.
(e) **Moderate-Intensity Continuous Training (MICT)**: Continuous aerobic work (walking, cycling) performed at 40–60% THR for 3–20 min bouts.
**Cooldown (5–10 min)**: Low-intensity activity, stretching, and guided breathing to promote recovery.

**Intensity prescription and monitoring **Training intensity was individualized using a combination of objective calculations and subjective feedback. THR was determined using the Karvonen formula [57], and strength loads were prescribed as percentages of the estimated one-repetition maximum (1RM). Estimated 1RM values were determined at baseline during the familiarization phase using submaximal strength testing and the Brzycki prediction equation [58], in accordance with established safety recommendations for older adults.

Although estimated 1RM was not formally reassessed during the 6-week intervention period, training loads were progressively adjusted throughout the program based on individual performance, repetition completion, and perceived exertion to maintain the intended relative intensity and ensure appropriate progressive overload. To facilitate real-time monitoring, calculated intensities were extrapolated to the modified Borg scale [59]. This load prescription and progression strategy followed the same methodology previously described in detail and validated in our earlier randomized crossover trial conducted in the same population [29].

### Functional performance assessments

Functional performance was evaluated at baseline and post-intervention using a battery of standardized tests recommended for the assessment of physical performance in older adults by recent international expert consensus [60] All assessments were conducted under controlled laboratory conditions by trained evaluators blinded to treatment allocation.

#### Short physical performance battery (SPPB)

The SPPB was administered as a composite measure of lower-extremity function using the validated Spanish version [30]. The battery consists of three subtests.

1. **Balance**: Participants attempted to maintain three stances of progressive difficulty (side-by-side, semi-tandem, and full tandem) for up to 10 s each.
2. **4-m Gait Speed**: The time (in seconds) to walk 4 m at a habitual pace was recorded.
3. **5-Repetition Sit-to-Stand (5R-STS)**: Participants performed five consecutive sit-to-stand cycles as quickly as possible without using their arms, and the total time was recorded.

Each subtest was scored from 0 (inability to perform) to 4 (best performance), yielding a total score of 0–12. The SPPB was included to ensure comparability with existing literature, acknowledging its potential for ceiling effects in high-functioning populations [30].

#### Timed up and go (TUG)

The TUG test assessed dynamic balance, mobility, and agility, as described in standardized protocols [60]. Participants rose from a standard chair, walked 3 m, turned around a cone, returned, and sat down as quickly and safely as possible. The total time to complete the task was recorded.

#### 400-Meter walk test

Walking endurance and cardiorespiratory fitness were assessed via the 400-meter walk test, following established methodology [60]. Participants were instructed to walk 400 m (10 laps of a 20-meter circuit) as quickly as possible without running. Standing rest was permitted, but sitting was not. The total completion time was recorded.

### Metabolic, Physiological, and inflammatory measures

All assessments were conducted in a temperature-controlled room at the same time of day pre- and post-intervention, following an overnight fast and 24-hour abstinence from alcohol and vigorous exercise, to minimize variability from circadian rhythms, nutrition, and physical activity.

#### Metabolic parameters

Metabolic parameters were assessed using a segmental multifrequency bioelectrical impedance analyzer (BIA; Tanita^®^ MC-580, Tokyo, Japan). While recognizing that indirect calorimetry represents the gold standard for measuring basal metabolic rate (BMR), BIA was selected for its practicality in field-based studies and its validated reliability for tracking longitudinal changes in metabolic parameters within individuals [61, 62].

The device applies dual-frequency currents (6.25 and 50 kHz) to estimate body composition and metabolic indices based on segmental impedance and demographic data. All measurements followed established guidelines: morning assessment after an overnight fast, bladder voiding, and standardized positioning [61].

The primary metabolic parameters derived from the BIA were:

- **Basal metabolic rate (BMR**,** kcal/day)**: Calculated by the device using predictive equations based on fat-free mass, which is the primary determinant of BMR.
- **Visceral fat index (scale 1–59)**: An estimate derived from the device’s proprietary analysis of trunk impedance data, providing a standardized indicator of abdominal adiposity.
- **Metabolic rate index**: A unitless score reflecting the individual’s measured BMR relative to the age- and sex-matched population average stored in the device’s database.
- **Metabolic age**: The chronological age corresponding to the population average for the participant’s measured BMR.

The Tanita MC-580 has demonstrated high test-retest reliability (coefficient of variation < 2%) in older adult populations, making it suitable for detecting intervention-induced changes despite its limitations in absolute precision compared to reference methods [62].

#### Physiological measures

Cardiovascular and respiratory parameters were assessed under standardized conditions following established clinical guidelines [63] All measurements were conducted in a quiet, temperature-controlled room at the same time of day pre- and post-intervention. Respiratory evaluations specifically adhered to the standards of the American Thoracic Society and European Respiratory Society [64].

The following parameters were recorded:

- **Blood Pressure and Heart Rate**: Resting systolic (SBP) and diastolic blood pressure (DBP) and heart rate were measured after 10 min of seated rest using an automated oscillometric sphygmomanometer (OMRON M6 Comfort, Omron Healthcare Co., Kyoto, Japan). Two readings were taken on the dominant arm with the cuff at heart level, and the mean value was used for analysis.
- **Peripheral Oxygen Saturation (SpO₂)**: Oxygen saturation was recorded via fingertip pulse oximetry (Nonin Onyx Vantage 9590; Nonin Medical Inc., Minnesota, USA) while participants remained seated, motionless, and breathing normally.
- **Maximal Expiratory Pressure (MEP)**: Expiratory muscle strength was assessed using a handheld digital manometer (MicroRPM, Micro Medical Ltd., Rochester, UK). Participants performed maximal expiratory efforts from total lung capacity against an occluded mouthpiece for at least 1.5 s. A minimum of three reproducible attempts (variability < 10%) was required, with the highest value (cm H₂O) recorded.

All measurements were performed by trained evaluators blinded to group allocation to ensure methodological consistency and minimize observer bias.

#### Inflammatory marker (Endothelial protein C Receptor, EPCR)

##### Rationale for biomarker selection

EPCR was selected as the primary inflammatory biomarker due to its dual role in vascular inflammation and endothelial activation. Unlike acute-phase reactants, EPCR reflects endothelial-specific inflammatory pathways that are particularly relevant in aging and exercise interventions [33, 34]. Its measurement provides insight into vascular inflammatory status, which may be modulated by combined exercise and nutritional supplementation.

##### Blood sampling and analysis

Fasting venous blood samples were collected after an overnight fast of at least 10 h. Samples were centrifuged at 3000 rpm for 10 min at 4 °C, and the resulting serum was aliquoted and stored at −80 °C until analysis.

Serum EPCR concentrations were quantified using a commercial enzyme-linked immunosorbent assay (ELISA) kit (Human EPCR ELISA Kit, Assay Genie, Dublin, Ireland; catalogue HUFI02847), following the manufacturer’s instructions. The assay sensitivity was 0.375 ng/mL, with a dynamic range of 0.625–40.625 ng/mL.

Values below the limit of detection (LOD = 0.375 ng/mL) were imputed as LOD/√2 (0.265 ng/mL), following established procedures for managing left-censored biomarker data [65]. This correction was applied to < 5% of samples. All samples were analysed in duplicate, with intra- and inter-assay coefficients of variation maintained below 10%.

### Safety and compliance

Safety and adherence were rigorously monitored throughout the trial using a multi-method approach. Weekly check-ins and structured monitoring forms documented supplement intake, exercise attendance, perceived well-being, and any potential adverse events. Supplement adherence was verified through returned sachet counts, participant logs, and direct supervision during exercise sessions. Exercise adherence was tracked via attendance sheets and training records.

#### Renal safety monitoring

Given the CRE supplementation, participants were specifically questioned about renal-related symptoms during weekly check-ins. No participants reported symptoms suggestive of renal impairment (e.g., edema, changes in urinary patterns). While formal renal function testing was not included in this short-term study, the absence of clinical symptoms and the established safety profile of low-dose creatine in healthy older adults support the safety of this intervention [50, 66].

No serious adverse events occurred during either supplementation phase or the exercise program. The only adverse effect reported was mild, transient muscle soreness during initial training sessions, which resolved spontaneously without intervention. Overall adherence exceeded 90% for both supplementation and exercise components, surpassing the pre-specified compliance threshold. While biochemical verification of supplement adherence was not performed, the combination of direct supervision, returned sachet counts (> 95%), and high exercise attendance provides strong evidence of protocol compliance.

### Statistical analysis

The primary endpoint for sample size estimation was gait speed, following EWGSOP2 recommendations [1]. A confirmatory family of functional performance measures (4-m gait speed, 5R-STS, TUG, and 400-m walk) were designated as co-primary outcomes. Secondary outcomes included metabolic/physiological parameters and inflammatory status (EPCR). Exploratory sex-specific analyses were conducted to examine potential differences in the response to the intervention; however, the study was not powered a priori to detect sex-by-treatment interactions.

#### Crossover design and carryover assessment

For the primary analysis, T1 and T3 values were pooled as pre-intervention (PRE), and T2 and T4 as post-intervention (POST). This approach, commonly used in crossover trials when no period or sequence effects are detected, increases statistical power and simplifies interpretation by focusing on the net treatment effect rather than phase-specific fluctuations. As a result, direct comparisons between individual phases were not performed, since the pooling strategy inherently integrates both treatment periods into a unified pre–post framework.

To rigorously assess potential carryover effects—a key consideration in crossover designs—we implemented multiple approaches: (I) direct comparison of PRE values at T1 and T3; (II) inclusion of treatment sequence (Placebo→CRE + HMB vs. CRE + HMB→Placebo) as a between-subject factor in ANCOVA models; and (III) formal testing of period and sequence effects for all outcomes (Table 4). These comprehensive analyses confirmed the adequacy of the 3-week washout period, supporting the validity of the pooled analysis.

**Primary Analysis Model**: The supplementation × time effects were evaluated using two-way repeated-measures ANCOVA models (time × condition), with age and the corresponding PRE value as covariates. This approach controls for age-related variability and baseline differences while enhancing sensitivity to detect within-subject treatment effects. Effect sizes were reported as partial eta squared (η²p) and interpreted according to established thresholds: η²*p* ≥ 0.01 (small), ≥ 0.06 (medium), and ≥ 0.14 (large) [63]. When significant interactions occurred, Bonferroni-adjusted post hoc tests were applied.

#### Secondary and exploratory analyses

Between-group differences at each time point were examined using univariate ANCOVA. Within-condition changes were assessed via one-way repeated-measures ANCOVA. To identify predictors of functional improvement, exploratory stepwise linear regression analyses were conducted using absolute change scores (Δ). Candidate predictors included supplementation condition, sex, and changes in metabolic/physiological parameters. Model diagnostics ensured collinearity (VIF < 2), residual independence, and homoscedasticity.

All analyses were performed using IBM SPSS Statistics v25, with statistical significance set at *p* < 0.05. Data are presented as mean (standard deviation), with normality verified using the Shapiro–Wilk test. Observed effect sizes for the primary outcomes (η²*p* = 0.17–0.42) exceeded the medium effect size assumed in the a priori power analysis, confirming adequate statistical power for the detected treatment effects.

## Results

At baseline (T1, prior to any intervention), no significant between-group differences were observed for any functional performance, metabolic, physiological, or inflammatory parameters (all *p* > 0.05; see Supplementary Table S1 for full baseline characteristics). Consequently, subsequent crossover analyses utilized pooled pre-intervention values (PRE: T1 and T3) and post-intervention values (POST: T2 and T4) to evaluate the treatment effect.

### Functional performance

At PRE (pooled T1/T3), no between-group differences emerged for any functional performance test (all *p* > 0.05). As anticipated in this high-functioning cohort, SPPB showed a marked ceiling effect, with the vast majority scoring 12/12, limiting discriminatory power at this stage. Main PRE and POST values by condition are presented in Table 1.

Significant time×group interactions were detected for 4-m gait speed, 5R-STS, TUG, and the 400-m walk in the total sample and in men (all *p* < 0.001), indicating superior improvement with CRE + HMB versus placebo. No interaction was observed for SPPB or static balance, consistent with ceiling effects.

At POST, the CRE + HMB phase outperformed placebo in gait speed, 5R-STS, TUG, and 400-m walk in the total sample and in men (all *p* < 0.05). Among women, POST differences favored CRE + HMB in gait speed, TUG, and 400-m walk (all *p* < 0.05), with no differences in SPPB or balance.

Within-phase changes corroborated these patterns: the CRE + HMB phase improved from PRE to POST across all functional outcomes in the total sample (SPPB, balance, gait speed, 5R-STS, TUG, 400-m; all *p* < 0.05). In men, all outcomes improved except balance; in women, all improved except SPPB. The placebo phase showed no significant within-phase changes.

Given SPPB ceiling effects, the primary inferences rely on sensitive mobility/endurance endpoints (gait speed, 5R-STS, TUG, 400-m). To mitigate potential carry-over, all inferential models adjusted for PRE values (pooled T1/T3), focusing on within-subject change.

**Table 1:** Functional physical performance parameters before and after supplementation with CRE plus HMB or placebo in older physically active adults

	Total Sample (*n* = 30)			Male (*n* = 20)			Female (*n* = 10)		
Group/ Period	CRE + HMB	CONTROL	*p* Ƞ^2^_*p*_	CRE + HMB	CONTROL	*p* Ƞ^2^_*p*_	CRE + HMB	CONTROL	*p* Ƞ^2^_*p*_
	SPPB Score								
PRE	11.67 (0.71)	11.70 (0.70)	0.096 0.052	11.70 (0.57)	11.75 (0.55)	0.095 0.079	11.60 (0.96)	11.6 (0.96)	0.624 0.017
POST	12.00 (0.01)$	11.70 (0.70)*		12.00 (0.00)$	11.65 (0.74)*		12.00 (0.00)	11.8 (0.63)	
	**Balance (Sec)**								
PRE	19.31(5.39)	21.51 (5.23)	0.409 0.016	18.98 (6.00)	20.62 (5.74)	0.714 0.005	19.98 (4.10)	23.30 (3.65)	0.278 0.125
POST	22.29 (4.28)$	22.89 (4.50)		21.28 (4.74)	21.93 (5.27)		24.31 (2.21)$	24.81 (0.63)	
	**Gait Speed 4 m (Sec)**								
PRE	2.68 (0.72)	2.54 (0.67)	**0.005** 0.157	2.67 (0.85)	2.53 (0.65)	**0.023** 0.156	2.68 (0.41)	2.57 (0.74)	0.130 0.177
POST	1.93 (0.37)$	2.40 (0.65)*		1.86 (0.34)$	2.38 (0.73)*		2.06 (0.40)$	2.45 (0.49)*	
	**5R-STS (Sec)**								
PRE	9.31 (2.40)	8.23 (3.05)	**< 0.001** 0.275	9.18 (2.39)	8.11 (2.48)	**< 0.001** 0.305	9.57 (2.52)	8.49 (4.10)	0.110 0.226
POST	6.16 (1.69)$	7.75 (3.02)*		5.94 (1.51)$	7.89 (3.04)*		6.60 (2.00)$	7.48 (3.14)	
	**TUG (Sec)**								
PRE	7.63 (1.26)	7.38 (1.28)	**< 0.001** 0.156	7.56 (1.33)	7.38 (1.10)	**0.002** 0.189	7.77 (1.17)	7.39 (1.66)	0.161 0.108
POST	6.29 (0.99)$	7.20 (1.46)*		6.16 (1.00)$	7.21 (1.65)*		6.54 (0.99)$	7.20 (1.09)*	
	**400 m Walk**								
PRE	3.60 (0.49)	3.41 (0.53)	**< 0.001** 0.294	3.62 (0.53)	3.42 (0.57)	**< 0.001** 0.381	3.57 (0.40)	3.40 (0.47)	0.071 0.183
POST	3.01 (0.49)$	3.33 (0.52)*		2.93 (0.51)$	3.31 (0.58)*		3.19 (0.42)$	3.36 (0.40)*	

Data are expressed as mean (standard deviation). Analyses were performed in the total sample (*n* = 30) and separately by sex (men: *n* = 20; women: *n* = 10)

*p*: Interaction effects (time × supplementation group) were examined using two-way repeated-measures ANCOVA, including chronological age and the baseline value as covariates. When appropriate, Bonferroni adjustments were applied for multiple comparisons

η²p: Partial eta squared, reported as the measure of effect size

*Indicates a significant difference between groups at the same assessment point (*p* < 0.05) based on univariate ANCOVA with group as the fixed factor, adjusted for age and PRE value

$Indicates a significant within-group change from baseline (*p* < 0.05) according to one-way repeated-measures ANCOVA (factor: time), adjusted for age and PRE value

PRE values correspond to assessments at T1 and T3, and POST values to T2 and T4

### Physiological, metabolic, and inflammatory outcomes

At PRE, no between-group differences were detected in physiological, metabolic, or inflammatory measures (all *p* > 0.05) (Table 2).

Significant time×group interactions were observed for BMR in the total sample (*p* < 0.001), metabolic rate index in the total sample (*p* = 0.026), DBP in women (*p* = 0.020), and EPCR in men (*p* = 0.021). Within-phase analyses showed significant improvements in MEP during the CRE + HMB phase for the total sample and specifically for women (both *p* < 0.05), with no significant changes during placebo.

Between-group POST comparisons indicated higher BMR and a favorable trend for lower visceral fat during CRE + HMB versus placebo. DBP and MEP improvements were more evident in women, suggesting potential cardiovascular and respiratory benefits. However, the reduction in diastolic blood pressure, although statistically significant (*p* = 0.015), was clinically modest (< 5 mmHg), indicating a mild physiological adaptation rather than a therapeutic effect. No significant effects were identified for SBP, heart rate, or SpO₂.

Analysis of the inflammatory biomarker EPCR revealed a sex-specific response. While the time×group interaction was not significant for the total sample (*p* = 0.920), a significant interaction in men reflected divergent changes between conditions. The modest rise in EPCR during the CRE + HMB phase remained within the physiological range and was not accompanied by adverse changes in blood pressure or systemic inflammation, suggesting a context of adaptive endothelial modulation rather than dysfunction.

**Table 2:** Physiological, metabolic, and inflammatory outcomes before and after supplementation with CRE plus HMB or placebo in physically active older adults

	Total Sample (*n* = 30)			Male (*n* = 20)			Female (*n* = 10)		
Group	CRE + HMB	CONTROL	*p* Ƞ^2^_*p*_	CRE + HMB	CONTROL	*p* Ƞ^2^_*p*_	CRE + HMB	CONTROL	*p* Ƞ^2^_*p*_
	SBP (mm Hg)								
**PRE**	129.10 (19.90)	131.10 (20.16)	0.794 0.001	131.70 (10.44)	132.60 (15.38)	0.707 0.005	123.90 (31.64)	128.10 (28.21)	0.992 < 0.001
**POST**	126.23 (16.83)	129.30 (18.20)		128.55 (13.84)	131.10 (12.65)		121.60 (21.74)	125.70 (26.60)	
	**DBP (mm Hg)**								
**PRE**	81.70 (9.23)	81.93 (9.62)	0.988 < 0.001	82.05 (6.71)	80.05 (7.46)	0.127 0.077	81.00 (13.37)	85.70 (12.53)	**0.015** 0.255
**POST**	80.50 (9.32)	80.77 (9.83)		79.90 (7.21)	81.55 (7.47)		81.70 (12.96)	79.20 (13.76)	
	** Heart rate (bpm)**								
**PRE**	71.07 (13.80)	72.43 (10.61)	0.505 0.010	70.75 (16.00)	71.35 (10.50)	0.634 0.010	71.70 (8.52)	74.60 (11.47)	0.451 0.016
**POST**	71.97 (13.59)	71.13 (11.28)		72.10 (14.98)	70.35 (12.58)		71.70 (11.02)	72.70 (8.49)	
	**SpO₂ (%)**								
**PRE**	96.53 (1.52)	96.53 (1.88)	0.606 0.004	96.45 (1.73)	96.45 (1.82)	0.872 0.001	96.70 (1.05)	96.70 (2.11)	0.319 0.050
**POST**	97.30 (1.34)	97.03 (1.56)		97.1 (1.51)	97.2 (1.19)		97.70 (0.82)	96.70 (2.16)	
	**MEP(cm H**_**2**_ **O)**								
**PRE**	391.67 (110.51)	408.33 (105.93)	0.309 0.021	442.50 (89.99)	426.25 (104.90)	0.798 0.002	290.00 (70.90)	332.50 (57.79)	0.090 0.185
**POST**	428.33 (109.01)$	426.67 (105.03)		478.75 (91.50)	476.25 (83.69)		327.50 (60.61)$	327.50 (66.09)	
	**BMR (Kcal/day)**								
**PRE**	1713.63 (301.50)	1724.63 (305.34)	**< 0.001** **0.180**	1885.94 (194.61)	1895.82 (204.11)	**0.009** **0.184**	1369.10 (122.94)	1382.20 (127.30)	**0.019** **0.234**
**POST**	1739.96 (308.70)	1719.26 (299.24)		1914.31 (204.12)	1888.50 (196.22)		1391.32 (128.44)	1380.71 (127.30)	
	**Visceral Fat Index**								
**PRE**	8.37 (3.89)	8.43 (3.96)	0.069 0.058	9.70 (3.84)	9.90 (3.82)	0.150 0.059	5.70 (2.40)	5.50 (2.32)	0.260 0.067
**POST**	8.13 (3.89)	8.60 (4.08)		9.45 (3.79)	10.10 (3.97)		5.50 (2.63)	5.60 (2.31)	
	**Metabolic Rate Index**								
**PRE**	11.07 (3.06)	11.07 (3.11)	**0.037** **0.065**	10.95 (3.26)	10.85 (3.34)	0.085 0.076	11.30 (2.75)	11.50 (2.71)	0.260 0.046
**POST**	11.43 (3.18)	11.00 (3.02)		11.30 (3.40)	10.70 (3.18)		11.70 (2.83)	11.60 (2.75)	
	**Metabolic Age**								
**PRE**	49.00 (11.63)	48.43 (10.70)	0.462 0.012	51.25 (11.52)	50.85 (10.64)	0.462 0.020	44.50 (11.05)	46.60 (9.54)	1.000 < 0.001
**POST**	48.33 (10.41)	48.47 (10.67)		50.25 (9.98)	50.90 (10.58)		44.50 (10.70)	43.60 (9.54)	
**EPCR (ng/mL)**									
**PRE**	40.50 (29.18)	55.17 (45.94)	0.920 < 0.001	39.27 (27.21)	66.69 (51.01)*	**0.044** **0.146**	42.93 (34.20)	32.12 (20.51)	0.745 0.007
**POST**	31.55 (48.92)	44.25 (40.46)		32.73 (50.93)	45.86 (42.53)		29.20 (47.18)	35.12 (42.10)	

Data are presented as mean (standard deviation). Analyses were performed in the total sample (*n* = 30) and separately by sex (men: *n* = 20; women: *n* = 10). SBP: systolic blood pressure; DBP: diastolic blood pressure; SpO₂: peripheral oxygen saturation; MEP: maximal expiratory pressure (cm H₂O); BMR: basal metabolic rate (kcal/day); EPCR: Endothelial Protein C Receptor

*p*: Interaction effects (time × supplementation group) were examined using two-way repeated-measures ANCOVA, including chronological age and the baseline (PRE) value as covariates. When appropriate, Bonferroni adjustments were applied for multiple comparisons

η²p: Partial eta squared, reported as the measure of effect size

Indicates a significant difference between groups at the same assessment point (*p* < 0.05) based on univariate ANCOVA with group as the fixed factor, adjusted for age and PRE value

$Indicates a significant within-group change from baseline (*p* < 0.05) according to one-way repeated-measures ANCOVA (factor: time), adjusted for age and PRE value.*

PRE values correspond to assessments at T1 and T3, and POST values to T2 and T4

Note: The modest increase in EPCR observed in men during the CRE + HMB phase remained within the expected physiological range and was not accompanied by adverse hemodynamic or inflammatory changes

### Predictors of functional performance

Exploratory regression models identified several independent predictors of functional performance (Table 3). The supplementation group (CRE + HMB) consistently emerged as a primary determinant of improved outcomes, particularly for gait speed, 5R-STS, and TUG, confirming the direct contribution of the intervention.

For balance, additional predictors included sex (better in women), higher BMR, lower metabolic age, lower DBP, and higher MEP. For 5R-STS, supplementation was the main predictor, with ΔSpO₂ providing additional support. For TUG, supplementation again predicted better performance, alongside lower metabolic age, higher BMR, and higher SpO₂. For the 400-m walk, lower metabolic age and higher BMR predicted faster times, while group allocation did not remain significant. Across models, adjusted R² ranged from 0.13 to 0.36, underscoring the clinical relevance of supplementation as the principal driver of functional improvements, complemented by the contribution of metabolic and physiological factors.

**Table 3:** Summary of linear regression models identifying predictors of functional performance outcomes in older physically active adults

Functional test	Predictors included	Standardized β	*p* value	Adjusted *R*²	Clinical interpretation
Balance (Equilibrium time)	Sex BMR Δ Metabolic age Δ DBP Group Δ MEP	−0.38 + 0.5 + 0.30 + 0.28 −0.30 + 0.24	< 0.001 < 0.001 0.009 0.015 0.014 0.031	0.357	Sex differences influenced balance. Higher BMR, lower metabolic age, reduced DBP, increased MEP, and supplementation improved balance.
Gait speed (4 m)	Group	−0.41	0.001	0.155	Intervention group achieved faster gait speed.
Sit-to-stand (5R-STS)	Group Δ SpO₂	−0.33 + 0.26	0.008 0.040	0.134	Intervention group performed 5R-STS faster; higher SpO₂ supported lower-body function.
Timed Up and Go (TUG)	Group Δ Metabolic age Δ SpO₂ BMR	−0.28 −0.36 0.25 −0.28	0.026 0.003 0.026 0.036	0.279	Intervention group achieved better TUG. Lower metabolic age and higher BMR supported mobility; higher SpO₂ also contributed.
400 m walk	Δ Metabolic age BMR	−0.43 −0.37	< 0.001 0.003	0.208	Reductions in metabolic age and higher BMR predicted faster 400 m walk (better aerobic capacity).

Data are presented as standardized beta coefficients (β), p values, and adjusted R² values derived from stepwise linear regression models. Candidate predictors included group assignment (CRE + HMB vs. placebo), sex, Δ metabolic and Δ physiological variables, and Δ metabolic age. Significant predictors reported in the table indicate variables that independently explained variance in each functional test. BMR: basal metabolic rate; O₂ saturation: resting oxygen saturation

### Assessment of carryover and period effects

Exploratory ANCOVA models were conducted to formally test for potential carryover and sequence effects by including period (T1 vs. T3), treatment sequence (Placebo→CRE + HMB vs. CRE + HMB→Placebo), and their interaction, adjusted for age. As detailed in Table 4, the sequence factor was not a significant source of variation for any functional, metabolic, physiological, or inflammatory outcome in the total sample or when stratified by sex. Although a small number of isolated period × sequence interactions reached statistical significance (e.g., for 5R-STS and 400-m walk), these effects were inconsistent across outcomes and sex strata and did not influence the primary findings related to supplementation. Collectively, these analyses support the adequacy of the 3-week washout period and confirm that carryover or order effects are unlikely to explain the observed benefits of supplementation.

**Table 4:** Exploratory ANCOVA testing the effect of period (T1 vs. T3), treatment sequence (Placebo→CRE + HMB vs. CRE + HMB→Placebo), and their interaction on functional outcomes, adjusted for age. Analyses were performed using pre-intervention values (T1 and T3)

Outcome	Effect	Total Sample (*n* = 30)			Male (*n* = 20)			Female (*n* = 10)		
		F	*p*	Partial η²	F	*p*	Partial η²	F	*p*	Partial η²
Functional physical performance parameters										
**SPPB score**	**Period (T1 vs. T3)**	7.608	0.008	0.122	6.152	0.018	0.149	2.360	0.145	0.136
	**Sequence**	0.006	0.936	0.000	0.030	0.864	0.001	0.094	0.763	0.006
	**Period × Sequence**	0.759	0.387	0.014	0.254	0.618	0.007	0.003	0.959	0.000
**Balance (s)**	**Period (T1 vs. T3)**	9.972	0.003	0.153	5.028	0.031	0.126	6.053	0.026	0.288
	**Sequence**	2.050	0.158	0.036	0.713	0.404	0.020	2.376	0.144	0.137
	**Period × Sequence**	1.898	0.174	0.033	2.352	0.134	0.063	0.090	0.768	0.006
**4-m Gait speed (m/s)**	**Period (T1 vs. T3)**	21.745	0.000	0.283	14.348	0.001	0.291	13.000	0.003	0.464
	**Sequence**	0.148	0.702	0.003	0.233	0.632	0.007	0.001	0.971	0.000
	**Period × Sequence**	1.502	0.226	0.027	0.059	0.810	0.002	9.572	0.007	0.390
** 5R-STS (s)**	**Period (T1 vs. T3)**	19.589	0.000	0.263	13.573	0.001	0.279	7.070	0.018	0.320
	**Sequence**	1.878	0.176	0.033	2.038	0.162	0.055	0.156	0.698	0.010
	**Period × Sequence**	4.945	0.030	0.082	1.823	0.186	0.049	1.037	0.325	0.065
**TUG (s)**	**Period (T1 vs. T3)**	14.025	0.000	0.203	7.271	0.011	0.172	6.255	0.024	0.294
	**Sequence**	0.234	0.630	0.004	0.140	0.711	0.004	0.041	0.843	0.003
	**Period × Sequence**	1.974	0.166	0.035	0.783	0.382	0.022	0.451	0.512	0.029
**400-m walk (min)**	**Period (T1 vs. T3)**	5.860	0.019	0.096	3.580	0.067	0.093	2.915	0.108	0.163
	**Sequence**	2.207	0.143	0.039	1.466	0.234	0.040	0.840	0.374	0.053
	**Period × Sequence**	7.386	0.009	0.118	2.829	0.101	0.075	5.547	0.033	0.270
**Physiological**,** metabolic**,** and inflammatory outcomes**										
**SBP (mm Hg)**	**Period (T1 vs. T3)**	0.012	0.914	0.000	0.003	0.959	0.000	0.006	0.937	0.000
	**Sequence**	0.151	0.699	0.003	0.042	0.839	0.001	0.166	0.689	0.011
	**Period × Sequence**	1.684	0.200	0.030	0.005	0.947	0.000	0.028	0.870	0.002
**DBP (mm Hg)**	**Period (T1 vs. T3)**	0.231	0.633	0.004	0.013	0.910	0.000	0.165	0.691	0.011
	**Sequence**	0.002	0.964	0.000	0.803	0.376	0.022	0.588	0.455	0.038
	**Period × Sequence**	0.247	0.621	0.004	0.004	0.951	0.000	0.117	0.737	0.008
**Heart rate (bpm)**	**Period (T1 vs. T3)**	1.820	0.183	0.032	2.505	0.123	0.067	0.037	0.850	0.002
	**Sequence**	0.095	0.759	0.002	0.005	0.945	0.000	0.422	0.526	0.027
	**Period × Sequence**	1.919	0.172	0.034	1.933	0.173	0.052	0.265	0.614	0.017
**SpO₂ (%)**	**Period (T1 vs. T3)**	5.251	0.026	0.087	3.638	0.065	0.094	1.260	0.279	0.078
	**Sequence**	0.049	0.826	0.001	0.007	0.932	0.000	0.050	0.825	0.003
	**Period × Sequence**	3.100	0.084	0.053	3.199	0.082	0.084	0.100	0.756	0.007
**MEP (cm H2O)**	**Period (T1 vs. T3)**	0.214	0.646	0.004	0.174	0.679	0.005	0.135	0.718	0.009
	**Sequence**	0.435	0.512	0.008	0.031	0.860	0.001	2.065	0.171	0.121
	**Period × Sequence**	0.127	0.723	0.002	0.180	0.674	0.005	0.130	0.723	0.009
**BMR (Kcal/day)**	**Period (T1 vs. T3)**	0.067	0.797	0.001	0.028	0.868	0.001	0.033	0.859	0.002
	**Sequence**	0.026	0.872	0.000	0.031	0.862	0.001	0.064	0.804	0.004
	**Period × Sequence**	0.586	0.447	0.011	0.001	0.971	0.000	0.288	0.599	0.019
**Visceral Fat Index**	**Period (T1 vs. T3)**	0.006	0.936	0.000	0.000	0.992	0.000	0.003	0.956	0.000
	**Sequence**	0.005	0.945	0.000	0.025	0.875	0.001	0.079	0.783	0.005
	**Period × Sequence**	0.470	0.496	0.008	0.008	0.929	0.000	0.679	0.423	0.043
**Metabolic Rate Index**	**Period (T1 vs. T3)**	0.012	0.914	0.000	0.129	0.721	0.004	1.168	0.297	0.072
	**Sequence**	0.000	0.988	0.000	0.012	0.914	0.000	0.173	0.684	0.011
	**Period × Sequence**	1.782	0.187	0.031	0.544	0.465	0.015	0.183	0.675	0.012
**Metabolic Age**	**Period (T1 vs. T3)**	0.071	0.791	0.001	0.022	0.884	0.001	0.247	0.627	0.016
	**Sequence**	0.099	0.754	0.002	0.029	0.867	0.001	0.247	0.627	0.016
	**Period × Sequence**	0.199	0.657	0.004	0.396	0.533	0.011	1.100	0.311	0.068
**EPCR (ng/mL)**	**Period (T1 vs. T3)**	13.269	0.001	0.194	15.452	0.000	0.306	2.933	0.107	0.164
	**Sequence**	2.057	0.157	0.036	7.262	0.011	0.172	1.753	0.205	0.105
	**Period × Sequence**	4.216	0.045	0.071	10.272	0.003	0.227	0.236	0.634	0.016

Period refers to comparisons between T1 and T3 (first vs. second intervention phase). Sequence refers to the treatment order (Placebo→CRE + HMB vs. CRE + HMB→Placebo). Period × Sequence denotes their interaction. All models were adjusted for age. Results are presented for the total sample (*n* = 30) and stratified by sex (males: *n* = 20; females: *n* = 10). Partial eta squared (η²p) is reported as effect size

Note: For EPCR, the significant period and sequence effects observed in men reflected modest within-range fluctuations without accompanying adverse hemodynamic or inflammatory changes, suggesting physiological variability rather than residual carryover or clinical alteration

## Discussion

### Main findings and interpretation

The present study demonstrates that six weeks of combined creatine plus β-hydroxy-β-methylbutyrate (CRE + HMB) supplementation, integrated into a supervised multicomponent training program, produced meaningful improvements in functional performance and metabolic efficiency in physically active older adults. These results expand current evidence supporting the complementary mechanisms of both supplements in enhancing muscular energetics, recovery capacity, and functional outcomes in aging populations [45, 50, 66].

Rather than reiterating well-established mechanisms of CRE and HMB, this discussion focuses on how their integration with structured multicomponent exercise translates into measurable improvements in gait speed, strength, and metabolic parameters. The magnitude of these effects—supported by large partial η² values (0.17–0.42)—indicates physiological and clinical relevance. Similar magnitudes of improvement have been reported in recent meta-analyses of CRE supplementation during resistance or multicomponent training in older adults [20, 42, 67].

From a translational perspective, the functional outcomes that showed the largest effect sizes—particularly gait speed, chair-stand performance, TUG, and walking endurance—are well-established indicators of mobility status and predictors of adverse health events in older adults [1–3, 31, 32]. Impairments in these measures have been associated with a higher risk of falls, mobility limitation, and loss of functional independence [2, 3, 31, 32]. Therefore, the magnitude of the observed effects likely reflects changes that extend beyond statistical significance and may be clinically meaningful, particularly in terms of mobility preservation and maintenance of independence in physically active older adults. Although clinical endpoints such as falls or hospitalizations were not directly assessed, the observed improvements in these functional domains suggest a potential for favorable downstream effects on clinically relevant outcomes that are known to be function-dependent in older adults.

Moreover, our findings align with current consensus guidelines emphasizing the integration of nutritional support and progressive exercise for the prevention of frailty and maintenance of functional independence in aging [56, 60]. The combined strategy tested here exemplifies a feasible and translational approach to enhance both muscular and metabolic resilience, complementing the growing body of literature supporting multimodal interventions for healthy aging [14, 15, 66].

### Integration with previous findings and overall interpretation

Building on these findings, the present study complements our previous crossover trial in the same population [29], which demonstrated improvements in muscle strength and endurance independent of mass gains. Together, both datasets provide a coherent picture of how CRE + HMB supplementation enhances functional capacity across multiple performance domains—strength, mobility, and metabolic health—in physically active older adults. This dual evidence reinforces the concept that CRE + HMB supplementation enhances muscle quality and functional efficiency, even in individuals without overt sarcopenia or frailty.

In line with this functional enhancement, the improvements observed in gait speed, 5R-STS , TUG , and the 400-m walk are clinically meaningful and align with previous studies reporting beneficial effects of CRE and HMB—administered individually or in combination with exercise—on functional capacity in older adults [18–21, 27, 28]. In contrast to studies limited to resistance training, our IPC model integrated resistance, endurance, balance, and coordination, providing a more comprehensive stimulus for both functional and metabolic adaptation.

The absence of significant changes in the SPPB likely reflects a ceiling effect associated with the high baseline functional status of the participants, rather than a lack of intervention efficacy. Ceiling effects in composite functional scales such as the SPPB have been widely reported in physically active or non-frail older adults, in whom baseline scores are often close to the upper limit of the scale, limiting the sensitivity of these instruments to detect further improvements following an intervention [68, 69]. In such contexts, additional physiological and neuromuscular adaptations may occur without translating into measurable changes in global performance scores. Accordingly, the lack of significant SPPB changes in the present study should be interpreted as a limitation of measurement sensitivity rather than as an absence of functional benefit.

From a study design perspective, these findings suggest that the SPPB may be more sensitive for detecting intervention effects in frailer populations or individuals with lower baseline function, where greater scope for improvement exists [70]. Conversely, future trials targeting higher-functioning older adults may benefit from incorporating more demanding or continuous functional assessments—such as extended gait tests, power-based measures, or endurance tasks—to better capture intervention-related adaptations.

According to partial eta-squared (η²p) thresholds proposed by Richardson (2011) [71], most functional outcomes—particularly gait speed, chair-stand performance, TUG, and the 400-m walk—showed large effect sizes (η²*p* = 0.15–0.29). These magnitudes confirm that the observed functional gains are not only statistically significant but also clinically meaningful, reinforcing the practical relevance of the intervention for maintaining mobility and independence in older adults.

### Mechanistic interpretation

The functional improvements observed in this study likely arise from convergent muscular, metabolic, and cardiovascular adaptations elicited by the combined CRE + HMB and exercise intervention. Both supplements act through complementary mechanisms that enhance muscle energetics and tissue efficiency. Creatine supports rapid ATP resynthesis through the phosphocreatine system, sustaining contractile performance and delaying fatigue during repeated efforts (for instance [20, 37, 42, 45, 50],. HMB, in turn, has been reported to attenuate proteolysis and enhance protein turnover and recovery, which may contribute to improved muscle integrity and functional performance in older adults [24, 27, 36]. Together, these actions are consistent with improvements in muscle quality—that is, greater functional output per unit of muscle—described in older populations [8]. The observed increases in BMR and metabolic rate index are compatible with an upregulation of energy turnover and bioenergetic efficiency during training and recovery. In older adults, CRE supplementation alongside resistance-type exercise has been associated with enhanced training adaptations (e.g., strength, lean mass), anabolic signaling, and muscle remodeling [19–21, 41, 42]and may modulate exercise-related oxidative and inflammatory responses [72]. These effects, integrated with supervised multicomponent training, provide a plausible physiological basis for the mobility improvements reported here [12, 14, 15, 55, 64].

Furthermore, the concomitant reductions in visceral fat index and diastolic blood pressure—particularly in women—suggest systemic metabolic and vascular benefits of the combined strategy. In women, the statistically significant yet clinically modest reduction in diastolic blood pressure (< 5 mmHg) likely reflects a mild enhancement in vascular compliance or autonomic regulation rather than a true antihypertensive effect. Prior evidence indicates that exercise-based programs in older adults can improve body composition and cardiometabolic health [12, 14–17, 55, 64] and attenuate low-grade inflammation linked to aging (“inflammaging”) [7, 72, 73]. Collectively, these findings support the concept that integrating targeted supplementation with structured multicomponent exercise can help sustain mobility and functional independence with aging [1, 10, 12, 14, 15].

In addition, the modest increase in circulating EPCR observed after the intervention should be interpreted with caution. While elevated EPCR may reflect enhanced endothelial turnover and adaptive remodeling secondary to exercise and improved perfusion, it can also indicate compensatory activation of the protein C pathway in response to subclinical vascular stress [33, 34]. The absence of concomitant rises in diastolic blood pressure or inflammatory markers, together with improved functional and metabolic parameters, supports the interpretation of this response as part of a favorable endothelial adaptation rather than endothelial dysfunction. While this adaptive interpretation appears most plausible, a transient pro-inflammatory response cannot be entirely ruled out. Nevertheless, future studies incorporating direct measures of vascular reactivity and soluble EPCR isoforms are warranted to clarify the precise clinical significance of this finding in aging populations.

Collectively, these findings illustrate that improvements in muscle energetics and vascular efficiency may underpin the enhanced functional outcomes observed. These physiological adaptations are further elaborated in Sect. 4.6, where mechanistic pathways underlying the observed synergy between CRE and HMB are discussed in greater depth.

### Duration and washout considerations

Regarding the methodological design, although reviewers highlighted the relatively short 6-week intervention and 3-week washout, several methodological and physiological arguments justify this design. First, previous trials have shown that moderate, loading-free CRE doses (3 g/day) achieve measurable functional and metabolic benefits within 4–6 weeks [27, 29, 42]. Second, under this dosing strategy, intramuscular creatine declines by approximately 1.6–1.7% per day after cessation, returning to baseline within 3–4 weeks [42, 43]. Third, formal analyses of potential carryover effects—including comparison of T1 vs. T3 values and ANCOVA models incorporating period and sequence terms (Table 4)—confirmed the absence of residual effects. These results, consistent with prior studies in older adults using similar protocols, validate the adequacy of the washout period and the robustness of the crossover design.

### Statistical and methodological robustness

In addition to this methodological rigor, the present analysis specifically addressed several statistical considerations raised by reviewers. Partial eta-squared (η²p) values were interpreted using established thresholds (small ≥ 0.01, medium ≥ 0.06, large ≥ 0.14) [71], providing a clearer understanding of the magnitude and practical relevance of the observed effects. The inclusion of Table 4 allowed formal testing for potential period, sequence, and carryover effects, thereby strengthening the internal validity and robustness of the crossover design. Furthermore, the regression analyses extended the inferential depth by identifying key metabolic (BMR, metabolic age) and physiological (SpO₂, DBP) predictors of functional gains.

Observed effect sizes for the primary outcomes (η²*p* = 0.17–0.42) were notably higher than the medium threshold assumed in the a priori power analysis, suggesting that the achieved sample size and statistical approach provided sufficient power to detect meaningful effects while minimizing type II error risk. This coherence between a priori estimates and observed effects supports the methodological soundness and reproducibility of the experimental design. Collectively, these results highlight the integrative nature of functional performance in aging, where metabolic efficiency and cardiorespiratory health jointly sustain mobility and independence.

### Comparison with previous literature

From a mechanistic perspective, both supplements act through complementary mechanisms that plausibly explain the observed synergy. Creatine increases intramuscular phosphocreatine availability, supports rapid ATP resynthesis, enhances high-intensity exercise capacity, and stimulates anabolic pathways such as mTOR and satellite cell proliferation [18, 21, 41]. HMB, on the other hand, attenuates proteolysis through inhibition of the ubiquitin–proteasome pathway, stabilizes cell membranes, and promotes recovery from exercise-induced muscle damage [24–26, 72] Together, these actions enhance muscle contractile quality and reduce fatigue during repeated submaximal efforts, leading to improved mobility and endurance. Furthermore, both nutrients have been linked to improved mitochondrial biogenesis, oxidative metabolism, and endothelial function [19, 25, 35] which may partly explain the concurrent improvements in BMR, oxygen utilization, and cardiopulmonary function observed here.

### Strengths, limitations, and future directions

Several limitations of the present study should be acknowledged. First, the relatively short intervention period (six weeks) limits the ability to assess the persistence of benefits or long-term adaptations following supplementation cessation. Second, body composition and basal metabolic rate were estimated using multifrequency bioelectrical impedance rather than gold-standard methods such as dual-energy X-ray absorptiometry or indirect calorimetry, which may reduce the precision of absolute values despite the method’s strong reliability for within-person changes. Third, the fixed dosing strategy (3 g/day of CRE and 3 g/day of HMB) was not normalized to body mass and could have contributed to interindividual variability, including the sex-specific differences observed. Moreover, biochemical verification of supplement adherence and single-supplement comparison arms (CRE-only or HMB-only) were not included, precluding a direct evaluation of additive versus synergistic effects. Although sex-related differences in fat-free mass (FFM) were evident, no statistical adjustment for this covariate was applied to avoid model overfitting given the modest sample size. These differences likely reflect physiological dimorphism rather than treatment-specific effects, yet future studies with larger cohorts should explore FFM-adjusted analyses to better disentangle compositional from functional influences. In addition, sex-specific analyses were exploratory in nature and the study was not powered a priori to formally test sex-by-intervention interactions. Furthermore, confidence intervals were not systematically reported for all effect size estimates, which may limit the precision of interpretation regarding the range of plausible effects. Furthermore, the high baseline functional status of the participants may have limited the sensitivity of the SPPB to detect further improvements, consistent with a ceiling effect in well-functioning older adults.

Nonetheless, several aspects mitigate these limitations: (i) the 3-week washout period exceeded the time generally required for reversal of exercise-induced functional gains in older adults; (ii) baseline equivalence (T1 vs. T3) and the absence of significant sequence effects (Table 4) support the adequacy of this control; and (iii) participants were instructed to maintain their habitual routines throughout the study, minimizing unsupervised variability. Collectively, these factors reinforce confidence that the observed effects primarily reflect the supplementation–exercise interaction rather than residual or external influences. Accordingly, no clinically relevant sequence or period effects were observed (Table 4), confirming the validity of the pooled PRE/POST analysis. Moreover, the training-induced baseline improvement across phases might have attenuated some between-condition contrasts.

Despite these constraints, this study presents several notable strengths. The randomized, double-blind, crossover design maximized statistical power and minimized interindividual variability. The intervention achieved excellent adherence (> 90%) and was conducted under direct supervision, ensuring methodological rigor and ecological validity. Moreover, the combined evaluation of functional, metabolic, and physiological outcomes provides a comprehensive understanding of the effects of CRE + HMB supplementation integrated with structured multicomponent training. Observed effect sizes exceeded a priori expectations, supporting the adequacy of the sample size and reinforcing the robustness of the study’s crossover design.

Future studies should extend these findings by incorporating longer follow-up periods, body mass–adjusted dosing, biochemical adherence verification, and advanced biomarkers of muscle metabolism and endothelial function. Such approaches will help clarify the mechanistic pathways and long-term sustainability of these benefits. Overall, the present results support the integration of targeted nutritional supplementation with multicomponent exercise as a feasible and safe strategy to enhance mobility, metabolic efficiency, and healthy aging in older adults.

## Conclusion

In summary, this randomized, double-blind, placebo-controlled crossover trial indicates that six weeks of combined creatine and β-hydroxy-β-methylbutyrate (CRE + HMB) supplementation, when integrated with a supervised multicomponent exercise program, produced meaningful improvements in functional performance, metabolic efficiency, and selected physiological outcomes in physically active older adults. These findings support the role of targeted nutritional strategies as potential enhancers of exercise adaptation, contributing to the maintenance of mobility and independence during aging.

These findings should be confirmed in longer interventions and in frail or sedentary populations to determine the durability and generalizability of these effects. Overall, CRE + HMB supplementation appears to be a safe, feasible, and practical adjunct to exercise-based interventions aimed at preserving muscle function and promoting healthy aging.

## Practical applications

The integration of creatine and β-hydroxy-β-methylbutyrate supplementation with structured multicomponent exercise offers a practical and evidence-based approach to promote functional independence and metabolic health in older adults. This combined strategy can be readily implemented in community or clinical settings using safe and cost-effective doses. From a clinical standpoint, the intervention may be particularly beneficial for older individuals aiming to preserve mobility, prevent frailty, and optimize the response to resistance or endurance training.

From a research perspective, further investigations should examine longer intervention periods, dose–response relationships, and underlying molecular adaptations—including mitochondrial function, oxidative stress, and circulating microRNAs—to better define the mechanisms contributing to functional and metabolic resilience during aging.

.

### Funding

This study did not receive any specific grants from funding agencies in the public, commercial, or not-for-profit sectors.

### Author contributions

R.R.-H. and J.M.-A. conceived and designed the study; oversaw data collection, analysis, and interpretation; and drafted the manuscript. A.M.-O., M.M.-F., M.S.-R., M.S.-P., and N.B. contributed to data acquisition, intervention supervision, and critical revision of the manuscript for important intellectual content. All authors participated in the interpretation of the results, reviewed and approved the final version of the manuscript, and agreed to be accountable for all aspects of the work.

## Supplementary Information

Below is the link to the electronic supplementary material.

Supplementary material 1 (DOCX 17.6 kb)

## Data Availability

The datasets generated and analyzed during the current study are available from the corresponding author on reasonable request.

## Declarations

### AI use declaration

Generative AI was not used to create or alter figures or data. Language editing assistance (if any) was performed under human oversight; authors take full responsibility for the content.
