# Correction: Combined creatine and β-hydroxy-β-methylbutyrate supplementation with integral conditioning exercise enhances functional performance and metabolic health in physically active older adults: A randomized controlled crossover trial

**DOI:** 10.1007/s40520-026-03377-5

**Published:** 2026-04-27

**Authors:** Rafael Ramos-Hernández, Natalia Busto, Álvaro Miguel-Ortega, María Martínez-Ferrán, Mirian Santamaría-Peláez, Miriam Saiz-Rodríguez, Juan Mielgo-Ayuso

**Affiliations:** 1 Faculty of Health Sciences, University of Burgos (UBU), Burgos, 09001 Spain; 2 IAFIV Research Group, University of Burgos (UBU), Burgos, 09001 Spain; 3 Faculty of Education, Alfonso X ‘El Sabio’ University (UAX), Madrid, 28691 Spain; 4 Working Group on Nutrition for Exercise and Sport, Spanish Nutrition Society “SEÑ”, Madrid, 28010 Spain

**Correction to: Aging Clinical and Experimental Research (2026) 38:44**

10.1007/s40520-025-03312-0

In the sentence beginning ‘During each 6-week intervention phase,’ in this article, the text ‘received either 3 g/day of CRE combined with 3 g/day of HMB (free acid form)’ should have read ‘received either 3 g/day of CRE combined with 3 g/day of calcium HMB (HMB-Ca)’.

The Original article has been corrected.

