# Supplementary material for: Combined creatine and β-hydroxy-β-methylbutyrate supplementation with integral conditioning exercise enhances functional performance and metabolic health in physically active older adults: A randomized controlled crossover trial
Source: Aging Clin Exp Res. 2026 Jan 9;38(1):44. doi: 10.1007/s40520-025-03312-0 (PMC12819432; doi:10.1007/s40520-025-03312-0)
Supplement: Supplementary file 1 — Supplementary material 1 (DOCX 17.6 kb) [file 40520_2025_3312_MOESM1_ESM.docx]

| **Supplementary Table S1.** Baseline (T1) characteristics of functional performance, quality of life, physiological, metabolic, and inflammatory parameters in older physically active adults supplemented with creatine plus HMB or placebo. | | | | | | | | | |
| --- | --- | --- | --- | --- | --- | --- | --- | --- | --- |
|  | **Total Sample (n=30)** | | | **Male (n=20)** | | | **Female (n=10)** | | |
| **Variable** | **CRE+HMB (n = 15)** | **Placebo (n = 15)** | **P** | **CRE+HMB (n = 10)** | **Placebo (n = 10)** | **P** | **CRE+HMB (n = 5)** | **Placebo (n = 5)** | **P** |
| **Functional physical performance parameters** | | | | | | | | | |
| **SPPB Score** | 11.53 ±0.87 | 11.36 ±0.93 | 0.647 | 11.55 ±0.69 | 11.50 ±0.71 | 0.853 | 11.50 ±1.22 | 11.00 ±1.41 | 0.867 |
| **Balance (Sec)** | 18.38 ±4.86 | 18.40 ±5.97 | 0.994 | 18.34 ±5.76 | 17.46 ±6.29 | 0.725 | 18.47 ±3.04 | 20.75 ±5.06 | 0.961 |
| **Gait Speed 4m (Sec)** | 2.91 ±0.80 | 3.04 ±0.64 | 0.651 | 3.06 ±0.92 | 2.92 ±0.69 | 0.674 | 2.65 ±0.49 | 3.36 ±0.43 | 0.156 |
| **5R-STS (Sec)** | 9.85 ±2.54 | 10.38 ±3.10 | 0.641 | 9.91 ±2.57 | 9.76 ±2.42 | 0.897 | 9.76 ±2.74 | 11.93 ±4.44 | 0.919 |
| **TUG (Sec)** | 7.92 ±1.36 | 8.21 ±1.40 | 0.613 | 7.88 ±1.42 | 8.00 ±1.24 | 0.787 | 7.99 ±1.39 | 8.71 ±1.84 | 0.961 |
| **400 Meter Walk** | 3.58 ±0.55 | 3.73 ±0.50 | 0.482 | 3.66 ±0.63 | 3.69 ±0.53 | 0.815 | 3.44 ±0.37 | 3.86 ±0.45 | 0.436 |
| **Physiological, metabolic, and inflammatory outcomes** | | | | | | | | | |
| **SBP (mm Hg)** | 125.82 ±17.07 | 134.71 ±24.90 | 0.272 | 131.64 ±10.82 | 132.50 ±19.10 | 0.832 | 115.17 ±22.09 | 140.25 ±39.18 | 0.702 |
| **DBP (mm Hg)** | 80.65 ±9.51 | 81.93 ±11.57 | 0.761 | 81.82 ±6.27 | 80.00 ±7.87 | 0.532 | 78.50 ±14.21 | 86.75 ±18.73 | 0.902 |
| **Pulse (bpm)** | 67.47 ±10.46 | 72.29 ±13.65 | 0.220 | 65.00 ±12.30 | 71.10 ±12.56 | 0.311 | 72.00 ±3.16 | 75.25 ±17.84 | 0.401 |
| **SpO₂ (%)** | 96.47 ±1.77 | 95.64 ±1.98 | 0.246 | 96.36 ±2.11 | 95.60 ±1.90 | 0.262 | 96.67 ±1.03 | 95.75 ±2.50 | 0.628 |
| **MEP (cm H_2_O)** | 394.12 ±122.00 | 417.86 ±78.10 | 0.491 | 447.73 ±113.17 | 450.00 ±63.46 | 0.908 | 295.83 ±64.06 | 337.50 ±47.87 | 0.224 |
| **BMR (Kcal/day)** | 1695.65 ±317.42 | 1768.64 ±281.21 | 0.522 | 1884.09 ±208.49 | 1906.60 ±180.76 | 0.874 | 1350.17 ±118.86 | 1423.75 ±150.33 | 0.646 |
| **Visceral Fat Index** | 8.06 ±4.49 | 8.93 ±3.00 | 0.596 | 9.82 ±4.64 | 9.80 ±2.66 | 0.964 | 4.83 ±1.47 | 6.75 ±2.99 | 0.650 |
| **Metabolic Rate Index** | 11.59 ±3.28 | 10.50 ±2.68 | 0.361 | 11.09 ±3.70 | 10.30 ±2.50 | 0.559 | 12.50 ±2.35 | 11.00 ±3.46 | 0.956 |
| **Metabolic Age** | 48.06 ±12.68 | 49.21 ±8.47 | 0.925 | 52.45 ±13.43 | 49.30 ±7.29 | 0.581 | 40.00 ±5.73 | 49.00 ±12.30 | 0.579 |
| **EPCR (ng/mL)** | 34.34 ±7.56 | 31.77 ±7.71 | 0.338 | 37.55 ±6.76 | 31.99 ±8.53 | 0.116 | 28.45 ±5.22 | 31.21 ±6.26 | 0.623 |
| Data are presented as mean ± standard deviation (SD). p: Between-group differences were assessed using ANCOVA with age as covariate. Abbreviations: SPPB, Short Physical Performance Battery; 5R-STS, Five-Repetition Sit-to-Stand; TUG, Timed Up and Go; SBP, systolic blood pressure; DBP, diastolic blood pressure; SpO₂, peripheral oxygen saturation; MEP, maximal expiratory pressure; BMR, basal metabolic rate; EPCR, Endothelial Protein C Receptor. | | | | | | | | | |
